# Informal carers’ health-related quality of life and patient experience in primary care: evidence from 195,364 carers in England responding to a national survey

**DOI:** 10.1186/s12875-015-0277-y

**Published:** 2015-05-15

**Authors:** Gwilym P.A. Thomas, Catherine L. Saunders, Martin O. Roland, Charlotte A.M. Paddison

**Affiliations:** Cambridge Centre for Health Services Research Primary Care Unit, University of Cambridge, Cambridge, UK; RAND Europe, Cambridge, UK; Department of Psychology, Anglia Ruskin University, Cambridge, UK

**Keywords:** Carers, Primary care, Health-related quality of life, Patient experience, Caregivers

## Abstract

**Background:**

We aim to describe the health-related quality of life of informal carers and their experiences of primary care.

**Methods:**

Responses from the 2011-12 English General Practice Patient Survey, including 195,364 informal carers, were analysed using mixed effect logistic regressions controlling for age, gender, ethnicity and social deprivation to describe carer health-related quality of life (mobility, self-care, usual activities, pain, and anxiety/depression, measured using EQ-5D) and primary care experience (access, continuity and communication).

**Results:**

Informal carers reported poorer health-related quality of life than non-carers of similar age, gender, ethnicity and social deprivation. Increasing caring commitment was associated with worse EQ-5D scores, with carers of 50+ hours a week scoring 0.05 points lower than non-carers (95 % CI 0.05 to 0.04), equivalent to 18 fewer days of full health annually. Considering each domain of EQ-5D separately, carers of 50+ hours/week were more likely to report pain OR = 1.53 (1.50-1.57), p < 0.0001, and anxiety/depression OR = 1.69 (1.66-1.73), p < 0.0001, than non-carers. Younger carers scored lower on EQ-5D than non-carer peers but the converse was true among over-85s. In the most deprived areas carers reported the equivalent of 37 fewer days of full health annually than carers in the most affluent areas. On average, carers reported poorer patient experiences in all areas of primary care than non-carers (odds ratios 0.84-0.97), with this difference being most marked in the domain of access.

**Conclusions:**

Informal carers experience a double disadvantage of poorer health-related quality of life and poorer patient experience in primary care. We find no evidence for health benefits of caregiving. We recommend physicians identify and treat carer health problems, including pain and anxiety/depression, particularly among young, deprived and high time-commitment carers. Improving patient experience for carers, including access to primary care, should be a priority.

**Electronic supplementary material:**

The online version of this article (doi:10.1186/s12875-015-0277-y) contains supplementary material, which is available to authorized users.

## Background

Promoting and protecting the health and wellbeing of informal carers is an important public health priority for both pragmatic and ethical reasons [1, 2], and the provision of high quality primary care services for carers is central to such efforts [3, 4].

Informal carers comprise between 10 and 30 % of the population of developed nations [5–7], and perform important social and economic roles [8, 9]. The economic value of informal caring has been estimated at £119 billion per year in the UK [10] and US$450 billion in the USA [11]. Informal care is set to take on an increasingly important role in supporting formal health services due to demographic changes associated with population aging and increasing financial pressures on healthcare [9, 12, 13].

Informal carers experience poorer physical and mental health [5, 8, 14–21] than non-carers. Greater care commitments, for example weekly time commitment or duration of caring responsibility [22, 23], are associated with increasingly poorer health. There is evidence that the burden of caring is most acute among marginalised groups, for example those who are socially isolated [24], or of lower socioeconomic status [25].

Recent analyses have suggested that caregiving is associated with lower mortality [26, 27], and some studies have suggested that low burden caring may benefit the health or wellbeing of the carer [28, 29]. Further empirical studies are needed to test for evidence supporting the ‘caring confers health benefit’” hypothesis and alternative explanations including the role of selection factors [30, 31] which determine who is able to undertake a caring role.

Previous research examining carers’ healthcare experiences has focused on their carer role [32]: the primary care experiences that carers report for themselves as *patients* remains unknown. Patient experience is an important dimension of care quality [33, 34], and knowledge of the patients’ experience can help to inform improvements in care.

This study aims to describe the health-related quality of life and primary care experiences of informal carers in England responding to the national General Practice Patient Survey, and to examine variation among carers reported by socio-demographic characteristics and level of caregiving commitment.

## Methods

The English General Practice Patient Survey (GP Patient Survey), a national primary-care based survey commissioned by the English Department of Health, is mailed annually to approximately 2.7 million patients who have been continuously registered with a general practice in England for at least six months. A random sample from each general practice in England is selected, stratified by age and gender; registered patients from practices which have typically had low response rates in previous years are over-sampled. Further details on the survey have been published [35–37].

### Measures

#### Caring

A single question was included in the GP Patient Survey from 2011 to identify informal carers, and measure caring commitment in terms of hours per week spent caring. Survey respondents were asked *‘Do you look after, or give any help or support to family members, friends or neighbours because of either: long-term physical or mental ill health/disability, or problems related to old age?”* Asked to discount anything they do as part of paid employment, respondents chose one of six response options: *No; Yes, 1–9 hours a week; Yes, 10–19 hours a week; Yes, 20–34 hours a week; Yes, 35–49 hours a week; Yes, 50+ hours a week.*

#### Health-related quality of life

Health-related quality of life was measured using the EuroQol five-dimension (EQ-5D) [38]. This standardised and well-validated measure [39] asks respondents to rate their health-related quality of life on five dimensions (mobility; self-care; usual activities; pain/discomfort; and anxiety/depression) with 3 response options corresponding to no, moderate, or severe problems for each dimension. Multiplied by 365, EQ-5D scores represent a standardized health utility score, interpretable as the number of days of full health experienced per year [39].

#### Patient experience

A single question was used to assess overall patient experience in primary care. Six additional items assessed patient experience in three domains: access (two questions); continuity of care (one question); and communication (receptionist communication (one question), doctor communication and, separately, nurse communication (one question with five sub-items for each)). Response options were provided on five- and six-point likert scales.

Patient experience outcomes were categorized using a binary indicator (yes/no) for ‘positive experience of care” consistent with the public reporting of GP Patient Survey data [40]. For questions on doctor and nurse communication we only included responses where at least three of the five sub-items were completed, coding an overall positive experience where all completed responses were either “very good” or “fairly good”. Detail on survey questions is provided in Additional file 1: Table S1.

#### Socio-demographic characteristics

Socio-demographic characteristics measured in the survey included self-reported gender (male/female); age (eight ordinal categories from 18–24, 25–34 to 85+); ethnicity using the UK Office of National Statistics categories of White; Mixed; Asian; Black; Other [41]) and socioeconomic status based on linking the respondent’s postal code to the Lower Super Output Area Index of Multiple Deprivation, a small-area measure of deprivation [42].

### Analysis

We described the characteristics of informal carers and non-carers who responded to GP Patient Survey by calculating weighted percentages. The associations between caring and health-related quality of life, and between caring and carer patient experience in primary care, were investigated using mixed effect regression models adjusted for age, gender, deprivation, and ethnicity using fixed effects, and additionally for primary care practice using a random effect. We compared respondents without caring commitments to carers, both overall and using five ordinal categories to investigate differences among carers by number of hours spent caring per week.

To explore whether the association between caring and health-related quality of life, or caring and carer patient experience, varied between different groups of carers, we carried out a further series of models which included interaction terms for age, gender, deprivation and ethnicity. We also explored the socio-demographic predictors of poorer health and poorer patient experience among carers alone.

For the analysis of health-related quality of life, we ran models which included each dimension of EQ-5D as a separate outcome. For patient experience outcomes only, as a sensitivity analysis, we explored whether the association between caring and patient experience could be explained by the poorer health-related quality of life of carers.

For one question we predicted adjusted percentages of carers and non-carers reporting a positive overall experience of primary care, these being the percentages we would expect to report a positive experience if the socio-demographic case-mix were the same as all included responders.

Multivariable analyses were carried out on respondents with complete data on socio-demographic characteristics and EQ-5D (855,330 responses including 174,035 carers). Stata 11 was used for all analyses. The GP Patient Survey is a service evaluation which does not require research ethics committee approval for its use.

## Results

1,037,946 responses to the 2011–12 English GP Patient Survey were received from patients registered with 8258 primary care practices (37.8 % survey response rate). 959,997 respondents provided a valid answer to the question about informal caring, of whom 195,364 (20.4 %) reported that they were informal carers, with 64,416 (33.0 %) indicating that their caring commitments exceeded 20 hours per week.

Demographic and health characteristics of the 195,364 respondents who self-reported as carers are displayed in Table 1. Carers were more likely to be older and female. Informal carers with higher caring time commitments were more likely to live in socially-deprived areas than carers with low time commitments.

**Table 1:** Demographic characteristics of patients with caring responsibilities among 2012 General Practice Patient Survey responders

	Self-reported carers (≥20 hours per week) N (weighted %) ^a^(total n = 64,416)	Self-reported carers (<20 hours per week)’ N (weighted %) ^a^(total n = 130,948)	Responders without caring responsibilities N (weighted %) ^a^(total n = 764,633)
**Gender**			
Male	25,466 (44.3)	51,736 (45.0)	336,635 (50.6)
Female	37,680 (55.7)	77,464 (55.0)	416,938 (49.4)
**Age group**			
18-24	1,141 (4.3)	3,506 (6.0)	40,138 (11.0)
25–34	3,061 (9.1)	7,266 (9.9)	90,015 (19.4)
35–44	6,789 (15.6)	14,824 (15.6)	114,513 (19.5)
45–54	11,300 (21.1)	32,379 (27.3)	128,503 (17.4)
55–64	15,309 (20.6)	38,906 (24.4)	142,044 (13.6)
65–74	13,958 (15.7)	22,711 (11.8)	131,974 (10.4)
75–84	9,418 (10.8)	8,328 (4.3)	79,584 (6.2)
85+	2,206 (2.8)	1,295 (0.7)	26,544 (2.5)
**Ethnic group**			
White	56,212 (87.9)	118,345 (90.7)	658,587 (86.9)
Mixed	404 (0.8)	775 (0.7)	5,967 (1.0)
Asian^b^	3,704 (6.4)	5,698 (5.2)	47,823 (6.7)
Black^c^	1,236 (2.1)	2,189 (1.7)	21,248 (2.8)
Other ethnic group	1,597 (2.8)	1,956 (1.6)	18,780 (2.5)
**Socio-economic deprivation**			
1 (Affluent)	9,516 (15.0)	30,424 (23.2)	147,459 (19.9)
2	11,764 (17.3)	30,626 (22.3)	155,496 (19.8)
3	13,141 (19.8)	27,911 (20.4)	156,665 (20.0)
4	13,683 (21.5)	22,850 (18.1)	150,733 (20.2)
5 (Deprived)	16,270 (26.6)	19,063 (16.0)	153,733 (20.1)
**Health-related quality of life**			
***Mobility:*** No problems	40,898 (70.4)	102,790 (83.4)	560,632 (81.8)
Some or severe problems	20,704 (29.6)	24,416 (16.6)	178,344 (18.2)
***Self-care:*** No problems	54,973 (90.4)	121,465 (95.9)	671,424 (93.0)
Some or severe problems	6,467 (9.6)	5,748 (4.1)	66,218 (7.0)
***Usual activities:*** No problems	39,545 (66.9)	98,595 (78.9)	555,514 (79.6)
Some or severe problems	22,160 (33.1)	28,780 (21.1)	183,891 (20.4)
***Pain/discomfort:*** None	25,386 (45.3)	68,766 (57.3)	419,714 (63.7)
Moderate or extreme pain/discomfort	36,021 (54.7)	58,100 (42.7)	316,938 (36.3)
***Anxiety/depression:*** None	39,076 (64.6)	94,086 (74.3)	556,103 (77.5)
Moderate or extreme anxiety/depression	21,093 (35.4)	31,424 (25.7)	170,633 (22.5)
**Number long-term conditions**			
0	22,039 (39.4)	57,657 (48.7)	351,412 (54.6)
1	19,155 (29.2)	41,460 (30.7)	218,265 (26.4)
2	12,075 (16.8)	19,540 (13.1)	106,832 (10.9)
3	6,335 (8.3)	7,959 (4.9)	51,457 (4.8)
4 or more	4,812 (6.3)	4,332 (2.6)	36,667 (3.3)

^a^ Weighted percentages are calculated using survey design and non-response weights (by age, gender, geographical location and GP practice, full details Technical Annex GP Patient Survey 2011–2012 Annual Report)

^b^ Indian, Pakistani, Bangladeshi, any other Asian background

^c^ Black Caribbean, Black African, any other Black background

### Health-related quality of life among carers

On average, carers reported poorer health-related quality of life that non-carers (weighted mean EQ-5D scores 0.81 and 0.84, respectively), with this difference interpretable as carers experiencing 11 fewer days of full health per year. Table 2 shows the difference in health-related quality of life for carers, compared to non-carers of similar age, gender, ethnicity, and level of social deprivation. Those with 50+ hours per week of caring commitment experience report worse health-related quality of life than non-carers (adjusted mean difference -0.05 (95 % CI-0.05 to-0.04)), interpretable as 18 fewer days of full health per year.

**Table 2:** Health-related quality of life (measured by EQ-5D) by number of hours caring per week

Caring commitment (hours/week)	Unadjusted mean EQ-5D (95 %CI)	Adjusted^a^ mean EQ-5D (95 %CI)
0	0.81 (0.81–0.81)	0.81 (0.81–0.81)
1–9	0.84 (0.84–0.84)	0.83 (0.83–0.83)
10–19	0.80 (0.80–0.81)	0.81 (0.81–0.81)
20–34	0.77 (0.77–0.78)	0.79 (0.78–0.79)
35–49	0.75 (0.75–0.76)	0.77 (0.77–0.78)
50+	0.73 (0.72–0.73)	0.77 (0.76–0.77)

^a^ Mean EQ-5D score after adjustment for gender, age, ethnicity, and social deprivation

**Fig. 1:**
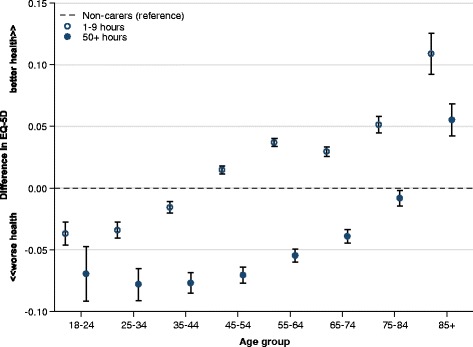
Difference in health-related quality of life among carers compared with non-carers, stratified by age; Health-related quality of life measured using EQ-5D

Carers aged under 45 score worse on EQ-5D than non-carers; those over 85 tend to score better (Fig. 1). Overall and within each age group, however, the effect of increasing caring commitment is a reduction in health-related quality of life.

Carers of 50+ hours/week were more likely to report pain OR = 1.53 (1.50-1.57), p < 0.0001, and anxiety/depression OR = 1.69 (1.66-1.73), p < 0.0001 than non-carers. When stratified by age, older carers at all levels of caring commitment were more likely to report pain, depression and anxiety, but reported better mobility and self-care ability than non-carers (Additional file 1: Figure S1).

For gender, deprivation and ethnicity, there was evidence of variation in the association between caring and health-related quality of life, although without consistent trends between groups. We therefore explored the association between socio-demographic characteristics and poorer health-related quality of life among carers alone (Table 3). This analysis showed differences in EQ-5D among carers by level of social deprivation and age, equivalent to 37 fewer days of full health per year for carers in the most deprived areas, compared to the most affluent, and 44 fewer days of full health among those aged 85+ compared with the 55-64 year old reference group.

**Table 3:** Differences in health-related quality of life among carers

	EQ-5D-predictors among carers(unadjusted)	p-value	EQ-5D-predictors among carers(adjusted)^a^	p-value
**Gender**				
Male	**ref**	<0.0001	**ref**	<0.0001
Female	0.01 (0.00 to 0.01)		0.00 (-0.01 to 0.00)	
**Age group**				
18–24	0.06 (0.05 to 0.07)	<0.0001	0.07 (0.06 to 0.08)	<0.0001
25–34	0.05 (0.05 to 0.06)		0.07 (0.06 to 0.07)	
35–44	0.03 (0.03 to 0.04)		0.04 (0.04 to 0.04)	
45–54	0.02 (0.01 to 0.02)		0.02 (0.01 to 0.02)	
55–64	**ref**		**ref**	
65–74	−0.03 (-0.03 to -0.03)		−0.02 (-0.03 to -0.02)	
75–84	−0.08 (-0.09 to -0.08)		−0.06 (-0.07 to-0.06)	
85+	−0.14 (-0.15 to -0.13)		−0.12 (-0.12 to -0.11)	
**Ethnic group**				
White	**ref**	<0.0001	**ref**	<0.0001
Mixed	−0.01 (-0.02 to 0.01)		−0.02 (-0.03 to 0.00)	
Asian	0.02 (0.01 to 0.02)		0.01 (0.01 to 0.02)	
Black	0.01 (0.00 to 0.02)		0.02 (0.01 to 0.03)	
Other ethnic group	−0.02 (-0.03 to -0.01)		−0.02 (-0.03 to-0.01)	
**Socio-economic deprivation**				
1 (Affluent)	**ref**	<0.0001	**ref**	<0.0001
2	−0.02 (-0.02 to -0.01)		−0.01 (-0.02 to -0.01)	
3	−0.03 (-0.04 to -0.03)		−0.03 (-0.03 to -0.03)	
4	−0.06 (-0.06 to -0.06)		−0.06 (-0.06 to -0.06)	
5 (Deprived)	−0.10 (-0.10 to -0.09)		−0.10 (-0.10 to -0.09)	

^a^ The results from this model are presented adjusted for level of caring commitment, age, gender, deprivation, ethnicity and general practice

### Patient experience among carers

Informal carers reported less positive patient experiences for all seven patient experience questions than non-carers, even after accounting for socio-demographic and health factors known to affect such experiences (odds ratios range: 0.84-0.97), with access the area where, compared with non-carers, patient experience is reported to be poorest. When asked a single question on overall experience in primary care, carers were between 1.0 and 3.7 % less likely (adjusted percentages, varying by level of caring commitment) to report a positive experience than non-carers.

**Fig. 2:**
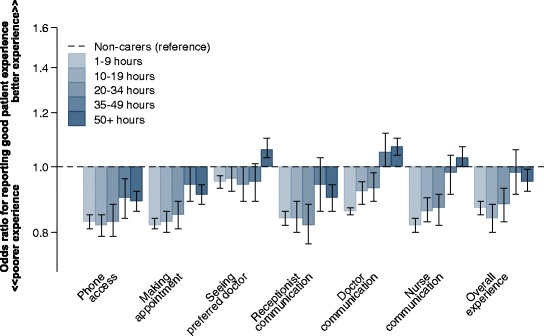
Variation in primary care experiences among people with self-reported carer responsibilities, by weekly care commitment

Among carers, there was some evidence of a trend showing that the likelihood of reporting a positive patient experience increased with higher levels of caring commitment (Fig. 2). Although all carers reported poorer access to healthcare (telephone access and making an appointment) than non-carers, those with caring commitments of over 50 hours per week rated their experiences more positively than non-carers in the domains of continuity and doctor communication (OR 1.07 (CI 1.03 to 1.1) and 1.04 (CI 1.01 to 1.07) respectively).

Controlling for health-related quality of life (EQ-5D) did not change the interpretation of observed associations between caring and patient experience (Additional file 2: Table S2).

In the same way as for health-related quality of life (Table 3), predictors of poorer patient experience among carers were explored (Table 4). Younger age was the strongest predictor of poorer patient experience among carers. Compared with the reference group of 55–64 years, the youngest carers (18–24 years) reported the poorest patient experience (OR 0.38 (CI 0.35 to 0.41)).

**Table 4:** Differences in patient experience among carers

	Unadjusted predictors of positive overall experience of care OR (95 %CI)	p-value	Adjusted predictors of positive overall experience of care OR (95 %CI)^a^	p-value
**Gender**				
Male	**ref**	p = 0.03	**ref**	p < 0.0001
Female	1.04 (1.00 to 1.07)		1.16 (1.12 to 1.20)	
**Age group**				
18–24	0.34 (0.32 to 0.37)	p < 0.0001	0.38 (0.35 to 0.41)	p < 0.0001
25–34	0.44 (0.41 to 0.47)		0.48 (0.45 to 0.51)	
35–44	0.62 (0.58 to 0.65)		0.64 (0.61 to 0.68)	
45–54	0.74 (0.71 to 0.78)		0.75 (0.72 to 0.78)	
55–64	**ref**		**ref**	
65–74	1.79 (1.69 to 1.90)		1.79 (1.69 to 1.90)	
75–84	2.61 (2.38 to 2.86)		2.61 (2.38 to 2.86)	
85+	2.34 (1.93 to 2.82)		2.35 (1.94 to 2.84)	
**Ethnic group**				
White	**ref**	p < 0.0001	**ref**	p < 0.0001
Mixed	0.55 (0.46 to 0.65)		0.80 (0.67 to 0.96)	
Asian	0.40 (0.37 to 0.42)		0.56 (0.52 to 0.59)	
Black	0.90 (0.80 to 1.02)		1.20 (1.06 to 1.36)	
Other ethnic group	0.64 (0.58 to 0.72)		0.88 (0.78 to 0.98)	
**Socio-economic deprivation**				
1 (Affluent)	**ref**	p < 0.0001	**ref**	p < 0.0001
2	0.55 (0.46 to 0.65)		0.98 (0.92 to 1.03)	
3	0.40 (0.37 to 0.42)		0.94 (0.89 to 1.00)	
4	0.90 (0.80 to 1.02)		0.89 (0.84 to 0.94)	
5 (Deprived)	0.64 (0.58 to 0.72)		0.86 (0.81 to 0.92)	

^a^ Positive patient experience defined as endorsement of “very good” or “fairly good” in response to question “Overall, how would you describe your experience of your GP surgery?” Odds ratios < .1.0 represent a poorer patient experience. The results are presented adjusted for level of caring commitment, age, gender, deprivation, ethnicity and general practice

## Discussion

Informal carers in England experience a double disadvantage of poorer health and worse patient experience in primary care when compared to non-carers of similar age, gender, ethnicity and level of social deprivation in a study of 195,364 carers responding to a national primary care-based survey.

### Health-related quality of life among carers

An association between caring and poorer health or quality of life is well-established from previous research [14, 15, 17, 23]. Our results add to what is already known by highlighting variation in this relationship by age; younger carers, particularly those aged under 45, reported poorer health-related quality of life than similarly-aged non-carers. The health needs of younger carers, which may be different from those of older carers [17, 43], can be identified as an important strategic priority for interventions aiming to improve carer health. In absolute terms, older carers nevertheless experience poorer health-related quality of life than younger carers, commensurate with their ageing.

### Health-related quality of life: evidence of benefits from caring?

Previous research has suggested that informal carers, particularly those with lower caring burdens, may experience health benefits from their role [26–29]; our results provide no additional evidence for this. Carers with higher caring time commitments report poorer health-related quality of life (EQ-5D) than those with lower time commitments, consistently across all age groups and the five dimensions of EQ-5D. However older carers in our study report better health-related quality of life than non-carers of similar age, particularly in the physical dimensions of EQ-5D. We suggest that this could be a consequence of carers often having to meet certain physical demands in order to assume a caring role; our results are more consistent with a process of self-selection based on physical capability making carers appear healthier [30, 31] than caring conferring significant social or psychological benefits.

### Disparities in health-related quality of life among informal carers

Carers living in the most deprived areas reported worse health-related quality of life than less deprived carers, even after controlling for other socio-demographic characteristics. This finding suggests that additional factors, such as available social capital, may be important in explaining disparities in health-related quality of life among carers. Consistent with this, previous studies have suggested that the presence of social support may reduce the burden experienced by carers [44] and can improve health outcomes for economically deprived individuals [45].

### Informal carers’ experiences as patients in primary care

Carers in the present study reported less positive patient experiences in primary care than non-carers, particularly for questions relating to access. Though the effects were not large in size, this pattern persisted even after socio-demographic characteristics known to influence patient experience were accounted for [35, 46, 47]. Findings suggest that problems with access to primary care among informal carers persist, even in a UK setting where a national health system with universal coverage enables access to free healthcare for all residents.

Further research is needed to explain the positive trend we observed between increasing caring commitment and better patient experience: we suggest increasing frequency of contact with family physicians among carers with a higher time commitment may be a contributory factor.

Among carers in our study, worse primary care experience was associated with being male, younger, non-white, and living in a socially deprived area, findings consistent with previous studies examining patient experience in primary care [47–49].

### Study considerations

Particular strengths of this study are the large sample size and use of data from a national primary care-based survey. Our findings contribute to existing literature in at least three important ways. First, this study is one of very few to provide information on the experiences of carers as *patients* in primary care (rather than in their role as carers for another patient). Second, our results highlight heterogeneity in quality of life among carers and identify younger carers as a priority. Finally, our results provide little empirical support for the hypothesis that small amounts of caring confer benefits to health-related quality of life for carers. We suggest future research considers how factors such as physical health influence the process of self-selection among potential caregivers, that is, potentially determining those who do, or do not, feel able to undertake a caring role.

This study also has some limitations. Data were collected through a national survey: we were reliant on respondents identifying themselves as informal carers, and no information on the nature of the caring relationship was available. In responding to survey questions, carers who encounter primary care in their capacity as a carer and, separately, as a patient, may have found it difficult to answer questions solely with their own experiences as a patient in mind. The response rate of 37.8 % is modest, though comparable with other large patient experience surveys [37, 50, 51]. Women and older people are over-represented among responders, and although people living in more deprived areas are less likely to respond overall, respondents come from all general practices in England, and all levels of income are included. It is possible that respondents in poorer health, or who have experienced poorer patient experience, are under-represented, but we would not expect this to be differential between people with and without caring responsibilities. Although we cannot be sure of the nature of response bias between different groups of carers, we note that in general response rates have been found only weakly to be associated with non-response bias in similar surveys [52–56]. Finally, the experiences of English carers in our study may differ from those of carers other countries due to differences in the cultural importance of caregivers, the structure of health and social care systems, and documented differences in carer burden [20, 57].

### Implications for policy and practice

Findings from this study have practical implications for the practice of family medicine, and for health policy. One component of any strategy to improve carer health should be to encourage primary care practices to continually review their patients and compile a “register” of those providing informal care. This would then enable family physicians to identify those with high caring commitments [58] and to provide proactive support, for example, using existing instruments to identify pain, anxiety and depression among carers, treating where appropriate [3, 4, 59]. Difficulties with this approach include the often gradual way in which people become carers, and the fact that they often do not identify themselves as such [58]. However, self-identification may be prompted by questions such as the one used in this study [36].

Interventions to improve carer health should consider evidence of heterogeneity among carers to ensure such interventions are targeted to those individuals who may benefit most and support is individualised. We recommend that family physicians focus on monitoring the overall health of younger carers and those living in deprived areas, and specific primary care interventions for these groups may need to be developed.

Improving the primary care experience of carers, particularly in relation to access, should be included as a strategic priority for health policy. A broad and coordinated approach from both policymakers and clinicians is needed in order to address the double disadvantage among informal carers of poorer health-related quality of life and poorer patient experience in primary care.

## Conclusions

Informal carers experience a double disadvantage of poorer health-related quality of life and worse patient experience in primary care when compared to non-carers of similar age, gender, ethnicity and level of social deprivation. There is heterogeneity among carers in terms of their health-related quality of life, with those who are younger, from deprived areas or who have high caregiving commitments experiencing fewer days in full health. We found no evidence from this work to suggest that caregiving confers health benefit on the carer; while carers over 80 years reported better health-related quality of life overall than non-carers of the same age, they scored worse on pain and anxiety/depression.

Our study was novel in investigating the primary care experience of informal carers as patients themselves, rather than focusing on their role as carer for another patient. Carers reported worse patient experience than non-carers, particularly in terms of access, with those carers who were male, younger, non-white, or living in a socially deprived area rating their experiences the most poorly.

We recommend that strategies to improve the wellbeing of informal carers focus on identifying caregivers, including those who are young or living in socially deprived areas, and address carers’ individual health needs including the treatment of pain and anxiety/depression. Improving the primary care patient experience for informal carers, particularly in terms of access, is an important priority.

## Additional files

Additional file 1: Table S1. Item content for seven questions from the General Practice Patient Survey 2012. **Figure S1.** The association between caring and health-related quality of life, presented separately for each domain of EQ-5D.

Additional file 2: Table S2. Likelihood of reporting a poorer overall patient experience among carers of different levels of caring commitment.

## Abbreviations

GP: General Practitioner/ General Practice
EQ-5D: EuroQuol five-dimension
UK: United Kingdom
OR: Odds Ratio
CI: Confidence Interval
UK: United Kingdom
