# Supplementary material for: Informal carers’ health-related quality of life and patient experience in primary care: evidence from 195,364 carers in England responding to a national survey
Source: BMC Fam Pract. 2015 May 15;16:62. doi: 10.1186/s12875-015-0277-y (PMC4446949; doi:10.1186/s12875-015-0277-y)
Supplement: Supplementary file 1 — Item content for seven questions from the General Practice Patient Survey 2012. Figure S1. The association between caring and health-related quality of life, presented separately for each domain of EQ-5D. [file 12875_2015_277_MOESM1_ESM.docx]

**Appendices**

**Appendix table 1. Item content for seven questions from the General Practice Patient Survey 2012**

| **Domain of care** | **GP Patient Survey item content** |
| --- | --- |
| Access | Generally, how easy it is to get to get through to someone at your GP surgery on the phone? |
| Access | Overall, how would you describe your experience of making an appointment |
| Continuity^a^ | How often to you see or speak to the GP you prefer |
| Communication (doctor) | Last time you saw or spoke to a GP, how good was that GP at each of the following? : giving you enough time; listening to you; explaining tests and treatments; involving you in decisions about your care; treating you with care and concern (five item composite) |
| Communication (nurse) | Last time you saw or spoke to a nurse, how good was that nurse at each of the following? : giving you enough time; listening to you; explaining tests and treatments; involving you in decisions about your care; treating you with care and concern (five item composite) |
| Communication (receptionists) | How helpful do you find the receptionists at you GP surgery? |
| Overall experience | Overall, how would you describe your experience of your GP surgery? |

a. Answered only by those who had a preference to see or speak to a particular doctor

**Appendix figure 1. The association between caring and health-related quality of life, presented separately for each domain of EQ-5D**

Results in **Appendix figure 1** are presented stratified by age (as in figure 1 of the main paper), for each domain of EQ-5D (Mobility, Self-Care, Usual Activities, Pain / Discomfort and Anxiety / Depression) separately. Hollow circles represent carers with a time commitment of 1-9 hours caring per week, and solid circles carers with a time commitment of 50+ hours of caring per week. The outcome for each figure is binary – defined as reporting some or severe problems for each domain of the EQ-5D. An odds ratio of less than one means that carers are more likely to report poorer health-related quality of life than non-carers of the same age.

These results show that carers report more pain and anxiety / depression than non-carers consistently across all age groups (except in the very old with low time commitment caring commitments) but carers report better physical functioning (mobility, self-care and usual activities) than non-carers at older ages. Higher caring commitments are associated with poorer outcomes than lower caring commitments at all ages and for all domains of care.

**Appendix table 2**. **Likelihood of reporting a poorer overall patient experience among carers of different levels of caring commitment.**

Odds ratios, adjusted for age, gender, deprivation, ethnicity and general practice, and then additionally adjusted for carer health

| **Caring commitment (hours/week)** | **Adjusted OR (95%CI) positive overall experience of care** | **OR (95% CI) additionally adjusted for EQ-5D** |
| --- | --- | --- |
| 0 | reference | reference |
| 1-9 | 0.88 (0.86 - 0.90) | 0.87 (0.85 - 0.89) |
| 10-19 | 0.84 (0.80 - 0.88) | 0.84 (0.80 - 0.88) |
| 20-34 | 0.87 (0.81 - 0.92) | 0.88 (0.83 - 0.93) |
| 35-49 | 0.95 (0.88 - 1.03) | 0.98 (0.91 - 1.06) |
| 50+ | 0.92 (0.88 - 0.96) | 0.95 (0.92 - 0.99) |
